# Navigating the Therapeutic Pathway and Optimal First-Line Systemic Therapy for Hepatocellular Carcinoma in the Era of Immune Checkpoint Inhibitors

**DOI:** 10.3390/medicina61122164

**Published:** 2025-12-04

**Authors:** Hyun Phil Shin, Moonhyung Lee

**Affiliations:** Department of Internal Medicine, Kyung Hee University Hospital at Gangdong, Kyung Hee University College of Medicine, 892 Dongnamro, Gangdong-gu, Seoul 05278, Republic of Korea; drmoon_0101@naver.com

**Keywords:** hepatocellular carcinoma, immune checkpoint inhibitors, anti-VEGF, adverse event

## Abstract

Hepatocellular carcinoma (HCC) remains a prevalent form of cancer with a poor prognosis and requires systemic therapies for most advanced cases. In this review, we summarize considerations for selecting treatment options for HCC, particularly with regard to immune checkpoint inhibitors (ICIs). Traditional chemotherapy has been surpassed by molecular-targeted therapies and ICIs, such as cytotoxic T-lymphocyte-associated antigen 4 (CTLA-4), programmed death-1 (PD-1), and programmed death-ligand 1 (PD-L1) inhibitors, which enhance the immune response against tumors. The European Association for the Study of the Liver (EASL) and American Association for the Study of Liver Diseases (AASLD) guidelines recommend atezolizumab/bevacizumab (Atez/Bev) and tremelimumab/durvalumab (Dur/Tre) as first-line treatments for unresectable HCC, along with alternatives, such as sorafenib and lenvatinib. Atezolizumab and bevacizumab have demonstrated superior efficacy but require the monitoring of bleeding risk and adverse events, such as proteinuria. Tremelimumab and durvalumab offer alternatives for patients at high risk of anti-Vascular Endothelial Growth Factor (anti-VEGF)-related complications. In cases where ICIs are contraindicated, lenvatinib and sorafenib serve as additional options, with lenvatinib demonstrating longer progression free survival (PFS) in clinical trials. It is important to consider that each treatment has specific side effects or contraindications, and the choice of medication should be based not only on the therapeutic efficacy of the drug, but also on the patient’s health status, liver function, and tumor characteristics.

## 1. Introduction

Hepatocellular carcinoma (HCC) is the sixth most common form of cancer with a high mortality rate [1]. HCC is highly prevalent in Asia compared to the West and is the third most common cause of cancer-related deaths in the Asia-Pacific region [2]. Despite continuous research and advancements in treatment methods that have gradually improved prognosis, the prognosis of patients remains poor with a median survival of less than two years [3]. Chronic hepatitis B infection remains the most prevalent etiology of HCC in Asia, except for Japan, followed by infection with hepatitis C virus (HCV) [4]. HCC primarily affects individuals with hepatitis or hepatitis-related cirrhosis, which may result from viral infections such as hepatitis B or C, excessive alcohol consumption, non-alcoholic steatohepatitis, or cirrhosis [5]. Therefore, treatment decisions should be guided by a multidimensional assessment that includes cancer stage, baseline liver function, patient performance status, and previous therapeutic exposures. Curative-intent treatments such as surgical resection, liver transplantation, and local ablation are typically viable only for early stages of liver cancer. However, most patients are diagnosed at an advanced stage of HCC, making curative treatment difficult. Advanced HCC was defined according to the BCLC staging system, corresponding to BCLC stage C, including patients with macrovascular invasion, extrahepatic spread, or those requiring systemic therapy [6]. These patients often present with tumor invasion into the blood vessels, bile duct, or extrahepatic metastasis, either at diagnosis or during treatment. However, advanced HCC differs from terminal cancer, as patients often maintain relatively good liver function and overall physical condition, thus allowing for the consideration of systemic therapies.

Systemic therapies for HCC include conventional cytotoxic chemotherapeutic agents, molecular-targeted therapies, and immune checkpoint inhibitor (ICI) therapies. However, cytotoxic chemotherapy is no longer utilized as a first-line treatment because no cytotoxic chemotherapy regimen has demonstrated superiority or non-inferiority to molecular-targeted therapies and ICIs, which are currently available options. Recently, multi-kinase inhibitors, such as sorafenib and lenvatinib, have been approved as first-line treatments for unresectable advanced HCC, providing improved survival benefits compared to cytotoxic chemotherapeutic agents, despite the specific adverse effects of each drug [7,8]. The landscape of HCC treatment has been notably transformed by the advent of immunotherapy [9,10].

## 2. ICI in HCC: Mechanisms and Clinical Implications

Immune checkpoint inhibitors (ICIs) are monoclonal antibodies that restore antitumor immunity by blocking inhibitory pathways such as PD-1/PD-L1 and CTLA-4. By preventing tumor-induced T-cell suppression, these agents enhance T-cell–mediated cytotoxicity and have emerged as an important therapeutic option for advanced HCC [11]. HCC tumors often exploit immune checkpoint pathways to avoid detection and destruction by the immune system. These checkpoints, which include specific inhibitory receptors and ligands, normally function to maintain immune balance and prevent autoimmunity. However, cancerous cells can manipulate these pathways to suppress the immune response, allowing tumors to grow unchecked by evading T-cell-mediated attack [12,13]. These antibodies function by blocking key regulatory signals that suppress the immune response, thereby enabling tumor-reactive T cells to effectively combat cancer, even in the immunosuppressive environment of the tumor microenvironment. This mechanism promotes the immune-mediated elimination of tumor cells, providing new hope in the battle against HCC [11]. The immune system recognizes antigens when a T-cell receptor (TCR) binds to an antigen on an antigen-presenting cell (APC). Immune checkpoints regulate this process to prevent autoimmunity [14]. Small retrospective studies suggest that atezolizumab plus bevacizumab may be used in CP-B patients, and grades 3–4 GI bleeding were reported in 16.7% and 10% of CP-B patients in Cheong’s and D’Alessio’s studies, respectively. Due to portal hypertension, the risk of variceal bleeding must be considered, it is crucial to review the bleeding risk, particularly in CP-B patients [15,16]. furthermore, tumors can exploit these checkpoints to evade immune detection [17]. ICIs disrupt these interactions by eliminating inhibitory signals and by enhancing the anti-tumor response of the immune system. The primary classes of ICIs target three main molecules: cytotoxic T-lymphocyte-associated protein-4 (CTLA-4), programmed cell death protein 1 (PD-1), and programmed death-ligand 1 (PD-L1).

Recent evidence indicates that CTLA-4 inhibitors exert functionality during the initial priming phase in lymphoid tissue, whereas PD-1 and PDL-1 inhibitors exert action during the effector phase in peripheral tissues [18]. T cell activation requires a co-stimulatory signal involving the cluster of differentiation 28 (CD28) on T cells and B7 on antigen-presenting cells. CTLA-4 competes with CD28 for B7 binding, thereby inhibiting the activation of T cells. CTLA-4 inhibitors enhance the activation of T-cells by augmenting CD28 and B7 co-stimulatory signals [19]. In lymphocytes, the expression of PD-1 increases after prolonged antigen exposure or during periods of lymphocyte exhaustion [20]. As with CTLA-4, PD-1 binding results in the reduced activation of T cells. However, PD-1 operates in peripheral tissues, unlike CTLA-4, which exerts functionality in lymphoid tissues. PD-L1, the ligand for PD-1, is commonly present in tumor cells, and its interaction allows tumors to escape immune detection. By blocking the interaction between PD-1 and PD-L1 in peripheral tissues, PD-1 and PD-L1 inhibitors can enhance the anti-tumor activity of T cells [21].

In this review, we focused on the application of first-line systemic therapies, particularly ICIs, and sought to facilitate the selection of first-line agents to maximize therapeutic efficacy while avoiding anticipated adverse events.

## 3. Overview of First-Line Systemic Therapies for HCC

Various guidelines recognize Atez/Bev and Dur/Tre as first-line systemic therapies for unresectable HCC [22], and additionally, the ESMO guideline indicates that nivolumab plus ipilimumab, durvalumab, or tislelizumab can be considered [23]. if these are not feasible, sorafenib and lenvatinib are recommended as alternatives [24,25,26,27]. First-line ICIs, such as Atez/Bev and Dur/Tre, have demonstrated superior efficacy over traditional tyrosine kinase inhibitors, such as IMbrave150 [28] and HIMALAYA [29].

### 3.1. Atez/Bev

Atezolizumab functions as an immune checkpoint inhibitor targeting PD-L1, whereas bevacizumab acts as an anti-Vascular Endothelial Growth Factor (anti-VEGF) agent, a pivotal protein that orchestrates angiogenesis and the formation of new blood vessels [28]. Combination therapy with Atez/Bev, which includes only one type of ICI, is favored as a long-standing first-line treatment. This poses less burden than the use of two types of ICIs: anti-CTLA-4 and anti-PD-L1 antibodies. The combination of anti-VEGF therapy with ICIs is designed to enhance drug delivery and reduce the requirement to reduce the risk of ICI-related toxicity [30].

In the IMbrave150 trial [28], median overall survival (mOS) was significantly longer in the Atez/Bev group than in the sorafenib group (19.2 months vs. 13.4 months; hazard ratio (HR), 0.66; *p* < 0.001) and PFS was also significantly longer than tin the sorafenib group (6.9 months vs. 4.3 months; HR, 0.65; *p* < 0.001). However, Atez/Bev treatment may cause side effects. In a previous study of 329 patients, 322 (98%) experienced treatment-related adverse events (trAEs), with 207 (63%) classified as grade 3 or 4 and 23 (7%) classified as grade 5; common trAEs included hypertension (29.8%), fatigue (20.4%), proteinuria (20.1%), and elevated liver enzyme (19.5%) [28] (Table 1).

Proteinuria, a significant adverse event in patients receiving VEGF monoclonal antibodies or anti-VEGF TKIs, is associated with a poor prognosis and reduced quality-of-life and requires careful management [31]. Fife et al. [8] reported a higher incidence of proteinuria in patients receiving Atez/Bev than in those receiving sorafenib (25% vs. 11%). Yang et al. [32] reported an increased risk of proteinuria with Atez/Bev than lenvima, especially in terms of macrovascular invasion. Ando et al. [31] found that initial impaired kidney function, taking blood pressure medications, and high systolic blood pressure (SBP) were risk factors for early proteinuria in patients receiving Atez/Bev therapy for unresectable HCC.

Although the frequency of bleeding is not extremely high, the fact that bleeding incidents occurred at a rate of 7% in the IMbrave150 trial [28] despite excluding high-risk patients, highlights a significant limitation in the use of Atez/Bev therapy. This highlights the need for the careful evaluation and management of gastroesophageal varices before prior to initiating therapy. Furthermore, bevacizumab is known to be associated with an increased risk of cardiac toxicity, thrombosis-related stroke, and gastrointestinal perforation, all of which require careful attention [11]. A recent study [33] indicated that hepatic decompensation significantly affected mortality in patients with HCC who treated with atezolizumab plus bevacizumab and that effective antiviral treatment reduced this risk.

### 3.2. Dur/Tre

#### STRIDE Regimen; Single Tremelimumab Regular Interval Durvalumab

The STRIDE regimen includes a single dose of tremelimumab in combination with durvalumab. This novel regimen is a combination of anti-CTLA-4 and anti-PD-L1 antibodies, and offers an alternative pathway for patients who are unable to tolerate anti-VEGF therapies owing to associated risks, such as bleeding and proteinuria [34].

In the HIMALAYA study [29], the STRIDE regimen significantly extended mOS (16.4 vs. 13.8 months; HR, 0.78; *p* = 0.0035) compared to sorafenib, although there was no significant difference in terms of PFS. The STRIDE regimen can also be challenging for patients owing to its known side effects; of the 388 patients (294 individuals) involved in this trial, 75.8% experienced treatment-related adverse events (trAEs), 25.8% of which were severe (grade 3 or 4). Nine deaths (2.3%) occurred during the study. Common trAEs included diarrhea (26.5%), pruritus (22.9%), rash (22.4%), reduced appetite (17.0%), fatigue (17.0%), pyrexia (12.9%), nausea (12.1%), increased AST levels (12.4%), and hypothyroidism (10.3%) (Table 1). Immune-related adverse events (irAEs) were clinically meaningful in the HIMALAYA study. 20.1% of patients required high-dose steroids for management, underscoring the need for close monitoring and timely intervention during treatment (Table 1). Hiroka et al. [35] documented that immunosuppressive therapies, including corticosteroid administration, were used in 21.9% of patients undergoing the STRIDE protocol, whereas hormone replacement therapies, excluding immunosuppressive interventions, were necessary in 2.2% of patients.

Despite the high incidence of irAEs requiring steroid use with Dur/Tre [35], this regimen is considered suitable for patients who need to maintain liver function, particularly those who are unable to continue anti-VEGF therapy. There is significant optimism about transitioning from one immunotherapy to another with less impact on the liver reserve.

### 3.3. Durvalumab Monotherapy

Durvalumab monotherapy, a selective PD-L1 inhibitor, provides an alternative therapeutic option for patients who are unable to tolerate anti-VEGF agents or combination immunotherapy [36].

### 3.4. Nivolumab Plus Ipilimumab (Nivo–Ipi Combination)

The Nivo–Ipi combination represents dual immune checkpoint blockade, targeting both PD-1 and CTLA-4 pathways to enhance antitumor immune activation. The combination of Nivolumab and Ipilimumab provides synergistic immune activation by concurrently inhibiting PD-1 and CTLA-4 signaling pathways [37]. Data from expanded analyses of the CheckMate studies have shown that this regimen can yield substantial antitumor activity, with meaningful objective response rates and durable survival benefits in selected patients with unresectable HCC [37]. In the cohort, this regimen demonstrated a notable objective response rate (31%) and durable responses in previously treated HCC patients, highlighting its potential role in the second-line setting [38]

### 3.5. Tislelizumab

Tislelizumab, a humanized IgG4 monoclonal antibody engineered to minimize Fcγ receptor binding, has emerged as a potential monotherapy for advanced HCC. Tislelizumab has gained attention as a potential alternative for patients unable to undergo anti-VEGF therapy or CTLA-4–containing regimens [39]. The RATIONALE-301 trial established Tislelizumab as non-inferior to Sorafenib (median OS 15.9 vs. 14.1 months; HR 0.85) and demonstrated a lower incidence of high-grade toxicities [39]. Its manageable safety profile and simplified dosing schedule support the potential role of Tislelizumab as a first-line option in patients requiring a more tolerable immune-based approach [40]. In the trial, tislelizumab achieved non-inferior overall survival compared with sorafenib (mOS, 15.9 vs. 14.1 months; HR 0.85) with a lower incidence of high-grade adverse events, supporting its role as a tolerable first-line option [41].

### 3.6. Lenvatinib

Lenvatinib is an oral multi-kinase inhibitor targeting the VEGF receptor (VEGFR), fibroblast growth factor receptor (FGFR), platelet-derived growth factor receptor (PDGFR), and other receptors such as rearranged during transfection (RET) [42,43]. In the REFLECT trial [8], lenvatinib exhibited a non-inferior mOS (13.6 vs. 12.3 months; HR, 0.92) compared to the sorafenib group. Furthermore, the PFS was also significantly longer in the lenvatinib group when compared to the sorafenib group (PFS: 7.4 vs. 3.7 months; HR, 0.66; *p* < 0.00001) (Table 1). This trial included patients with HCC involving less than 50% of the liver volume, without invasion of the bile duct or main portal vein, and with preserved liver function, classified as Child-Pugh(CP)-A.

In the REFLECT trial [8], the most common trAEs in the lenvatinib group were hypertension (42%), diarrhea (39%), reduced appetite (34%), and hand-foot skin reaction (HFSR, 26.9%), whereas those in the sorafenib group were HFSR (52%), diarrhea (46%), hypertension (30%), and decreased appetite (27%). Although lenvatinib and sorafenib are associated with similar trAEs, the lenvatinib group featured fewer cases of HFSR but more cases of hypertension than the sorafenib group (Table 1). Importantly, despite superior median progression-free survival and higher objective response rates compared with sorafenib, lenvatinib did not demonstrate an overall survival advantage, indicating limitations in long-term disease control [44]. Moreover, lenvatinib is generally not recommended for patients with Child–Pugh class B liver function because of reduced tolerability and limited efficacy evidence in this population [45,46].

### 3.7. Sorafenib

Sorafenib is a multi-tyrosine kinase inhibitor that targets the receptor tyrosine kinase activity of VEGFR, PDGFR, serine-threonine kinase Raf-1, and B-raf [47,48]. In 2007, sorafenib was the first molecular-targeted agent to prove survival benefit in advanced cases of HCC. In the SHARP (Sorafenib Hepatocellular Carcinoma Assessment Randomized Protocol) trial, mOS was significantly longer in the sorafenib group than in the placebo group (10.7 months vs. 7.9 months; HR 0.69, *p* ≤ 0.001); furthermore, median time to radiological progression was significantly longer in the sorafenib group than in the placebo group (5.5 vs. 2.8 months; HR, 0.58; *p* < 0.001) (Table 1). All phase 3 clinical trials of systemic therapies for advanced HCC were conducted in patients with CP-A, and the patients included in the SHARP trial were rated as CP-A (97%) at baseline. However, a previous uncontrolled phase 2 study of sorafenib [49] involving hepatoma patients with CP-A or B status, indicated that single-agent sorafenib may exert a beneficial therapeutic effect. In the GIDEON (Global Investigation of Therapeutic Decisions in Hepatocellular Carcinoma and Of its Treatment with Sorafenib) study [50], sorafenib was more effective for both CP-A and CP-B. The most common irAEs in the sorafenib group were diarrhea (39%), fatigue (22%), and HFSR (21%) (Table 1). Of these, HFSR can lead to treatment delay or a reduced quality-of-life. Furthermore, Dranitsaris et al. [51] reported that female gender, good performance status, and multiple metastases were predictors for HFRS.

## 4. Selecting Optimal First-Line Agents

### 4.1. Atez/Bev

A direct comparison between the Atez/Bev and Dur/Tre regimens has yet to be conducted. In the IMbrave150 study, Atez/Bev exhibited a PFS of 6.8 months and a response rate of 27.3%. Conversely, the HIMALAYA trial reported a PFS of 3.8 months and a 20.1% response rate for Dur/Tre; moreover, the rate of disease progression was lower with Atez/Bev (19.6%) than with Dur/Tre (39.9%). Unlike the HIMALAYA trial, the IMbrave150 trial included high-risk patients with invasion of the main portal vein or bile duct, or at least 50% liver involvement. Hatanaka et al. [52] suggested that Atezolizumab plus Bevacizumab may be preferred for patients with a high tumor burden, such as those with ≥50% liver involvement or those with portal vein tumor thrombosis. Kudo [53] suggested that for locally advanced HCC (irrespective of vascular invasion) without massive extrahepatic spread, an immuno-oncology (IO) plus anti-VEGF regimen is preferred because of its broad benefits and lower toxicity. Kuwano et al. [54] reported that tremelimumab and durvalumab are more effective for HCC patients with inactivated WNT/β-catenin signaling and high CD8+ TILs. Therefore, Atez/Bev is preferred as a first-line systemic therapy option for patients without ICI contraindication, particularly for patients with a higher risk of irAEs and locally advanced HCC (with or without vascular invasion).

### 4.2. STRIDE Regimen (Dur/Tre)

Atez/Bev therapy poses risks such as proteinuria, especially in those with kidney issues or high blood pressure, and a significant bleeding risk, requiring careful management in patients with portal hypertension. Additionally, bevacizumab may lead to cardiac toxicity, stroke, and gastrointestinal perforation, necessitating vigilant monitoring. For patients at high risk of anti-VEGF-associated complications, initiating treatment with a combination of tremelimumab and durvalumab as a first-line option is gaining notable traction [29].

### 4.3. Durvalumab Monotherapy

Durvalumab monotherapy represents a clinically viable option for patients who cannot receive anti-VEGF therapy or for whom combination immunotherapy is not appropriate [55]. In the HIMALAYA trial, Durvalumab demonstrated non-inferior OS compared with Sorafenib (16.6 vs. 13.8 months; HR 0.86), accompanied by a favorable toxicity profile with fewer high-grade immune-related adverse events [55]. These characteristics suggest that Durvalumab is suited to patients in whom preservation of hepatic reserve is essential, particularly those at risk for complications related to VEGF-R inhibition or CTLA-4–based therapy [56].

### 4.4. Nivo–Ipi Combination

Nivo–Ipi Combination has demonstrated superior OS compared to sorafenib/lenvatinib in patients with unresectable HCC in a recent phase III trial [53]. Additionally, the duration of response was higher than with lenvatinib or sorafenib. High burden of grade ≥ 3 immune-related toxicities necessitates careful clinical judgment, as patients with marginal liver function or autoimmune predisposition may be less suitable for such intensive immunomodulation [55]. But it presents a different frequency of complications compared to the STRIDE regimen (PD-1 and CTLA-4 regimen), tremelimumab may be used as an alternative dual ICI regimen based on these differences.

### 4.5. Tislelizumab

Tislelizumab has shown non-inferior survival versus sorafenib in first-line unresectable HCC and is now being evaluated globally as a potential first-line ICI option [39]. Its manageable safety profile and simplified dosing schedule support the potential role of Tislelizumab as a first-line option in patients requiring a more tolerable immune-based approach [40].

### 4.6. Sorafenib and Lenvatinib

In current guidelines [6,23,57,58], combinations including at least one PD-1 or PD-L1 inhibitor are recommended, provided there are no contraindications. However, targeted therapies in HCC, such as sorafenib and lenvatinib, play a crucial role as first systemic therapies for patients who cannot use ICIs. These therapies work by inhibiting angiogenesis and various molecular pathways. Lenvatinib has advantages over sorafenib, including superior mPFS and fewer complications like HFSR. Sorafenib can be used in patients with CP-B liver function.

## 5. Conclusions

Systemic therapies with atezolizumab plus bevacizumab or durvalumab plus tremelimumab have been recognized as preferred first-line treatment options by several recent guidelines [6,59]. These combinations are considered for patients without contraindications to ICI therapy, such as those with autoimmune diseases or those who have undergone stem cell or solid organ transplantation. According to guidelines [6,23,57,58], at least one PD-1 or PD-L1 inhibitor should be offered, provided there are no contraindications. The combination of atezolizumab and bevacizumab is associated with an improved mOS and mPFS when compared to sorafenib. However, it is crucial to assess the risk of anti-VEGF-related complications, such as gastrointestinal bleeding before treatment. The combination of durvalumab and tremelimumab (the STRIDE regimen) is a viable alternative for patients at high risk of gastrointestinal bleeding, such as those with esophageal varices identified via esophagogastroduodenoscopy (EGD), or those with significant proteinuria.

While the durvalumab plus tremelimumab (STRIDE) regimen is not associated with the same anti-VEGF inhibitor-related adverse events, the addition of anti-CTLA-4 to anti-PD-L1 has raised concerns relating to the higher incidence of irAEs; furthermore, the STRIDE regimen has been associated with organ failure in up to 75% of liver transplant recipients.

In scenarios in which ICI-based therapy is not feasible, alternatives, such as sorafenib or Lenvatinib, may be utilized. Sorafenib was the first agent to demonstrate a survival benefit for advanced HCC; however, lenvatinib has been associated with a non-inferior mOS with a significantly extended PFS and a lower incidence of HFSR than sorafenib.

**Figure 1:**
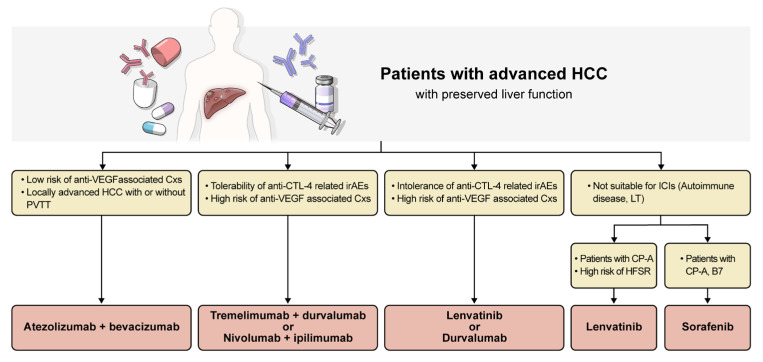
Treatment algorithm of 1st line systemic therapy for advanced hepatocellular carcinoma. CP, Child-Pugh score; CTL-4, anti-cytotoxic T-lymphocyte-associated protein-4; Cxs, complications; HCC, hepatocellular carcinoma; HFSR, hand-foot skin reaction; ICIs, immune checkpoint inhibitors; irAEs, immune-related adverse events; LT, liver transplantation; PVTT, portal vein tumor thrombosis; VEGF, vascular endothelial growth factor.

Deciding the appropriate first-line systemic treatment requires careful consideration of the patient’s liver function, tumor burden, and comorbidities (Figure 1). With the progression of ICI therapies, outcomes superior to those of traditional treatments are expected, and ongoing research is likely to yield more approved options.

## 6. Integration of Systemic Therapy into Curative-Intent Strategies

Traditional treatment algorithms have divided patients with HCC into considered suitable for surgical resection and requiring systemic therapy. However, contemporary guidelines now emphasize that patients with initially unresectable disease may become candidates for resection or liver transplantation if they achieve adequate tumor control with systemic or locoregional therapies [60].

Systemic therapy may reduce tumor burden sufficiently to allow downstaging or conversion to resection in selected patients. When the future liver remnant (FLR) is inadequate for safe hepatectomy, portal vein embolization (PVE) is widely used to increase FLR volume, and PVE combined with stem cell–based strategies (PVESA) has been investigated to further enhance FLR growth and expand surgical eligibility [61]. Conversely, in patients who undergo curative resection or ablation, the routine use of adjuvant Atez-Bev is not currently recommended due to unclear benefit-to-risk profile [62].

## 7. Future Directions

All Phase 3 trials of first line systemic therapy focus on CP-A patients; only limited evidence is available for patients with CP-B. However, real-world studies of sorafenib [50] have reported comparable TTP and safety profiles between CP-A and CP-B. Sorafenib represents a potential option for HCC patients with CP-B, with careful monitoring of liver-related side effects. Recent studies have reported that lenvatinib and other mTKI agents are also safe for patients with CP-B liver disease. However, two retrospective studies of lenvatinib [63,64] have reported results with a mOS of 17.8 months and 21 months for the CP-A group but 8.8 months and 9 months for the CP-B group, respectively. Studies on CP-B patients treated with ICIs have revealed poor outcomes, with a median survival of 6 to 6.7 months for those receiving atezolizumab and bevacizumab [52,65]. Retrospective data [66] further suggest that single-agent anti-PD1 or anti-PD-L1 therapies may represent viable options for patients with CP-B liver disease.

Numerous studies confirm poor survival rates for CP-B patients, with a lower mOS than CP-A. Current data do not support unrestricted use of approved therapies for CP-B, thus highlighting the need for well-designed trials to build a robust evidence base for this group [67].

## Figures and Tables

**Table 1:** Patient-centric comparison of first-line systemic therapies for HCC.

Regimen	Definition	Description	Mechanism	Advantages	Disadvantages	Adverse Effects
Atezolizumab + Bevacizumab	Combination of ICI targets PD-L1 (Atezolizumab) and anti-VEGF monoclonal antibody (Bevacizumab)	Established first-line regimen based on IMbrave150 with survival benefit.	PD-L1 blockade enhances T-cell activity; VEGF inhibition improves immune infiltration.	Better both mOS and mPFS than sorafenib. IMbrave 150 included high risk patients such as main portal vein, bile duct. Invasion.	Risk of bleeding, proteinuria; requires endoscopic evaluation.	Hypertension, proteinuria, fatigue, GI bleeding.
Durvalumab + Tremelimumab (STRIDE)	Dual ICI, targets PD-L1 (4 week-interval Durvalumab) and CTLA-4 (Single priming dose of Tremelimumab)	The HIMALAYA trial evaluated the dual ICI, so-called STRIDE regimen vs. sorafenib Patients with the risk of anti-VEGF associate Cxs can be considered	CTLA-4 inhibition (priming phase) + PD-L1 inhibition (effector phase).	Improved mOS vs. sorafenib without anti-VEGF-associated complications	PFS was not statistically significantly different. Higher rate of immune-related AEs; requires close monitoring. Not studied in HCC with main portal vein invasion in HIMALAYA HIMALAYA trial, HCV-related HCC: the least benefit	Diarrhea, rash, pruritus, hepatitis, hypothyroidism.
Durvalumab (monotherapy)	Single ICI targets PD-L1	The HIMALAYA trial sorafenib vs. single agent durvalumab Patients with the risk of anti-VEGF or dual ICI regimen-associated Cxs.	Blocks PD-L1/PD-1 interaction.	OS was noninferior with durvalumab monotherapy compared to sorafenib. Lower toxicity; feasible in fragile patients.	Lower ORR compared with combination therapy.	Fatigue, pruritus, hypothyroidism.
Nivolumab + Ipilimumab	Dual ICI targets PD-L1 (Nivolumab) and CTLA-4 (Ipilimumab)	In the CheckMate 9DW trial, Nivolumab plus ipilimumab showed a significant overall survival benefit versus lenvatinib or sorafenib	PD-1 inhibition + CTLA-4 inhibition.	Duration of response was higher than lenvatinib or sorafenib.	Higher immune-related toxicities. Limited real-world data	Colitis, hepatitis, dermatitis, endocrinopathies.
Tislelizumab	Single ICI targets PD-1	In the RATIONALE-301, non-inferior OS to sorafenib Patients with the risk of anti-VEGF or dual ICI regimen associate Cxs.	PD-1 inhibition revitalizes T-cell response.	Monotherapy option when use of dual ICI contraindicated. Fewer TRAEs leading to discontinuation and fewer grade 3 TRAEs than sorafenib. ESMO	Limited real-world data. RATIONALE 301 trial, HBV-related HCC: the least benefit.	Fatigue, infusion reactions, immune-mediated AEs.
Lenvatinib	Oral multi-kinase inhibitor.	REFLEC trial, compared with sorafenib	Targets VEGFR, FGFR, PDGFR, RET.	Superior mPFS; lower risk of HFSR; higher response rate.	Not suitable for CP-B patients; no OS advantage over sorafenib.	Hypertension, diarrhea, appetite loss, HFSR (less frequent than sorafenib).
Sorafenib	Oral multi-kinase inhibitor approved for HCC.	SHARP trial, standard-of-care first line therapy.	Targets RAF, VEGFR, PDGFR.	Proven over a long period-long-term safety profile. Usable in CP-B7	Lower response rate; QoL impact from HFSR.	HFSR, diarrhea, fatigue.

AEs, adverse events; CP, Child-Pugh; CTLA-4, cytotoxic T-lymphocyte-associated protein-4; ESMO, European Society for Medical Oncology; FGFR, fibroblast growth factor receptor; HFSR, hand–foot skin reaction; ICI, immune checkpoint inhibitor; mOS, median overall survival; mPFS, median progression-free survival; PD-1, programmed death-1; PD-L1, programmed death-ligand 1; PDGFR, platelet-derived growth factor receptor; STRIDE, single tremelimumab regular interval durvalumab; VEGF, vascular endothelial growth factor; VEGFR, vascular endothelial growth factor receptor.

## Data Availability

Data sharing is not applicable to this article as no new data were created or analyzed in this study.

## Abbreviations

AASLD	American Association for the Study of Liver Diseases
AE	Adverse event
APC	Antigen-presenting cell
AST	Aspartate aminotransferase
Atez/Bev	Atezolizumab plus bevacizumab
BCLC	Barcelona Clinic Liver Cancer
CD28	Cluster of differentiation 28
CP	Child–Pugh
CTLA-4	Cytotoxic T-lymphocyte-associated protein-4
Dur/Tre	Durvalumab plus tremelimumab
EASL	European Association for the Study of the Liver
EGD	Esophagogastroduodenoscopy
FGFR	Fibroblast growth factor receptor
HCC	Hepatocellular carcinoma
HFSR	Hand–foot skin reaction
HR	Hazard ratio
ICI	Immune checkpoint inhibitor
IMbrave150	Phase III trial of atezolizumab plus bevacizumab
irAE	Immune-related adverse event
IST	Immunosuppressive treatment
mOS	Median overall survival
mPFS	Median progression-free survival
mRP	Median time to radiologic progression
mTKI	Multi-tyrosine kinase inhibitor
NASH	Non-alcoholic steatohepatitis
PD-1	Programmed death-1
PD-L1	Programmed death-ligand 1
PDGFR	Platelet-derived growth factor receptor
PFS	Progression-free survival
PVTT	Portal vein tumor thrombosis
RET	Rearranged during transfection
RCT	Randomized controlled trial
SBP	Systolic blood pressure
SHARP	Sorafenib Hepatocellular Carcinoma Assessment Randomized Protocol
STRIDE	Single tremelimumab regular interval durvalumab
TCR	T-cell receptor
TKI	Tyrosine kinase inhibitor
TTP	Time to progression
VEGF	Vascular endothelial growth factor
VEGFR	Vascular endothelial growth factor receptor
